# Sex Disparities in Hepatitis C Treatment Initiation: Role of Universal Access to DAA in France (ANRS FANTASIO 2)

**DOI:** 10.1111/liv.70884

**Published:** 2026-09-18

**Authors:** Fabienne Marcellin, Clémence Ramier, Cécile Brouard, Vincent Di Beo, Yoann Allier, Abbas Mourad, Morgane Bureau‐Stoltmann, Philippe Sogni, Sylvie Deuffic‐Burban, Patrizia Carrieri, Benjamin Rolland, Marc Bourliere, Camelia Protopopescu

**Affiliations:** ^1^ Aix Marseille Univ Inserm, IRD, SESSTIM, Sciences Economiques & Sociales de la Santé & Traitement de l'Information Médicale, ISSPAM Marseille France; ^2^ Santé publique France Saint Maurice France; ^3^ ANRS | Maladies Infectieuses Emergentes Paris France; ^4^ Université Paris Descartes Paris France; ^5^ INSERM U1223 Institut Pasteur Paris France; ^6^ Service d'Hépatologie, Hôpital Cochin, Assistance Publique – Hôpitaux de Paris Paris France; ^7^ Université Paris Cité and Université Sorbonne Paris Nord Inserm, IAME Paris France; ^8^ PSYR, CNRL, INSERM U1028, CNRS UMR5292, UCBL1 Bron France; ^9^ Université Claude Bernard Lyon 1 Lyon France; ^10^ Centre Hospitalier Le Vinatier Bron France; ^11^ Service Universitaire d'Addictologie de Lyon (SUAL) HCL, CH Le Vinatier Lyon France; ^12^ Hôpital St Joseph Service d'Hépato‐Gastroentérologie Marseille France

**Keywords:** direct‐acting antivirals, elimination, healthcare databases, hepatitis C

## Abstract

**Background and Aims:**

Ensuring access to direct‐acting antivirals (DAA) for all individuals concerned is a priority in hepatitis C elimination efforts. We analysed sex disparities in DAA initiation in France over a 9‐year period spanning before and after August 2017, when the country implemented universal DAA access.

**Methods:**

We used data from the French National Health Data System for two periods: 2014–2017 and 2017–2022. Socioeconomic and clinical characteristics of adults identified with chronic hepatitis C and residing in metropolitan France were described at each period entry date. DAA initiation incidence rates were then estimated and multivariable Cox proportional hazards models were used to identify potential sex disparities for each period.

**Results:**

The study population comprised 97 817 and 98 020 individuals (61.3% and 62.0% men) before and after universal access to DAA, respectively. Comorbidity prevalence was significantly lower among women, except for psychiatric disorders. Compared with men, the incidence rate of DAA initiation was lower in women before universal access (24.5 [24.2–24.9] vs. 26.1 [25.8–26.3] per 100 person‐years (PY), *p* < 0.001), and higher after (22.3 [21.9–22.6] vs. 18.5 [18.3–18.7] per 100 PY, *p* < 0.001). These differences were confirmed in multivariable models.

**Conclusions:**

In France, universal DAA access has reversed sex disparity in treatment initiation among people with chronic hepatitis C. This reversal likely reflects differences in disease progression profiles between men and women. To ensure truly equitable access to HCV care, current research should explore whether reversal or reduction of sex disparities is observed in other countries and subpopulations.

## Lay Summary

In August 2017, France made direct‐acting antivirals (DAA) available without restriction to everyone with hepatitis C to improve equity in access to care. Before this policy, men were more likely than women to start DAA treatment, largely because of sex differences in disease progression risk factors. Universal access has helped close this gap, and even reversed sex difference in treatment initiation.

## Key Points

- In 2017, France made direct‐acting antivirals (DAA) available without restriction to everyone with hepatitis C.
- Universal DAA access has reversed sex disparities in hepatitis C treatment initiation.
- This change likely reflects differences in disease progression profiles between men and women.

**Abbreviations**
AERLI: training and education about safer injection *(Accompagnement et Education aux Risques Liés à l'Injection)*
aHR: adjusted hazard ratio
AME: state medical assistance *(Aide médicale de l'État)*
ATC: Anatomical Therapeutic Classification
CCMA: Common Classification of Medical Acts
CI: confidence interval
CSS: complementary state health insurance *(Complémentaire santé solidaire)*
DAA: direct‐acting antivirals
HBV: hepatitis B virus
HCV: hepatitis C virus
ICD: International Classification of Diseases
LTD: long‐term disease
NMBA: Nomenclature of Medical Biology Acts
OST: opioid substitution therapy
PAF: population attributable fractions
PY: person‐years
SNDS: French National Health Data System (*Système National des Données de Santé*)
WHO: World Health Organization

## 1 Introduction

Since the identification of hepatitis C virus (HCV) in 1989 [1], considerable progress has been made to treat chronic HCV infection (called chronic hepatitis C), which currently affects 50 million individuals worldwide [2]. Specifically, since 2013, interferon‐free direct‐acting antivirals (DAA)—which have a markedly better safety profile and a shorter treatment duration compared to interferon‐based therapies [3]—have helped cure almost all treated patients.

Previous studies on sex disparities in DAA efficacy and safety showed no clinically significant difference, although in some populations (e.g., with HCV genotype 1 or 3), women exhibited marginally higher cure rates and reported a slightly higher number of moderately severe treatment side effects compared with men [4]. Other research has highlighted sex disparities regarding DAA initiation rates for specific populations, including people who inject drugs [5] and persons with HIV‐HCV co‐infection [6].

In France, where chronic hepatitis C prevalence in persons aged 18–75 years is estimated at 0.34% [95% confidence interval (CI): 0.14–0.84] in men and 0.26% [0.05–1.21] in women [7], national surveillance data from 2014 (date of DAA arrival in the country) to 2021 showed sex disparities in hepatitis C screening, the majority of tested individuals being women. Furthermore, women under 39 years of age accounted for more than two‐fifths of all tested individuals [8]. By contrast, between 2015 and 2023, men accounted for the majority of individuals receiving care for chronic hepatitis C in France [9, 10]. In terms of specific populations, sex disparities were also found in access to hepatitis C treatment during the early DAA era (i.e., 2014 to 2016); for example, a lower proportion of women who inject drugs initiated therapy compared with their male counterparts [11].

In line with the World Health Organization (WHO) recommendations [12], which aim for the elimination of hepatitis C as a public health threat by 2030, France set the elimination of hepatitis C by 2025 as a national public health objective [13]. To achieve this target, universal access to DAA for all individuals with chronic hepatitis C was introduced on 3 August 2017, regardless of previous eligibility criteria which included fibrosis stage, the presence of comorbidities, and the presence of risk factors for HCV transmission. The present study aimed to explore potential sex disparities in adult DAA initiation before (i.e., 2014–2017) and after (i.e., 2017–2022) the key public health measure of universal access to DAA was implemented in metropolitan France.

## 2 Materials and Methods

### 2.1 Data Source

We analysed information from the French National Health Data System, called the SNDS (*Système National des Données de Santé*), which contains pseudonymized individual data on healthcare reimbursements for all people affiliated to the national social security system, representing more than 97% of the French general population [14, 15]. The SNDS also includes data on long‐term disease (LTD) status, an administrative measure which provides free treatment and care for people with certain chronic diseases, including hepatitis C [16], as well as data on medical causes of death.

### 2.2 Identification of People With Chronic HCV Infection

People with chronic hepatitis C were identified in the SNDS using a standard algorithm developed by clinical and technical experts on French health administrative databases [17]. This algorithm combines reimbursement data for treatment dispensation, biological tests, LTD status, and hospitalizations for chronic hepatitis C (Table 1). It was validated against the clinically established viral status in a French prospective cohort linked to the SNDS and showed good performance for identifying patients with chronic HCV infection (sensitivity 0.94 and specificity 0.85) [18].

**TABLE 1:** Algorithm used for identifying individuals with chronic hepatitis C in the French National Health Data System [17] (ANRS FANTASIO 2 study).

At least one criteria during year *N*		ATC codes
New LTD registration	ICD‐10 code B18.2	(pegylated interferon) L03AB04, L03AB05, L03AB09, L03AB10, L03AB11, L03AB60, L03AB61; (ribavirin) J05AP01; (boceprevir) J05AP03; (daclatasvir) J05AP07; (dasabuvir) J05AP09; (glecaprevir+pibrentasvir) J05AP57; (ombitasvir+paritaprevir+ritonavir) J05AP53; (simeprevir) J05AP05; (sofosbuvir) J05AP08; (sofosbuvir+ledipasvir) J05AP51; (sofosbuvir+velpatasvir) J05AP55; (sofosbuvir+velpatasvir+voxilaprevir) J05AP56; (telaprevir) J05AP02
Hospital claims for main or related diagnosis of hepatitis C	ICD‐10 code B18.2	
Dispensing of pegylated interferon and ribavirin at the same date		
Dispensing of direct‐acting antivirals for HCV		
Determination of HCV genotype	NMBA code 4125	
≥ 3 laboratory diagnostic tests for HCV RNA detection and quantification	NMBA code 4124	
**OR**		
≥ 2 laboratory diagnostic tests for HCV RNA detection and quantification **AND**	NMBA code 4124	
○ ≥ 1 examination for the assessment of liver fibrosis and/or	NMBA codes 1000, 1001, 1002, and CCMA codes HLQM002, HLBH001, HLHH001, HLHH005, HLHJ003	
○ Active LTD and/or	ICD‐10 code B18.2	
○ Hospital claims for associated diagnosis of hepatitis C	ICD‐10 code B18.2	

Abbreviations: ATC, Anatomical Therapeutic Classification; CCMA, Common Classification of Medical Acts; ICD, International Classification of Diseases; LTD, long‐term disease; NMBA, Nomenclature of Medical Biology Acts; SNDS: French National Health Data System (“Système National des Données de Santé”).

### 2.3 Study Periods and Study Populations

We conducted separate analyses for the following two time periods: 1 January 2014 to 3 August 2017 (study period 1: ‘before universal access to DAA in France’) and 4 August 2017 to 31 December 2022 (study period 2: ‘after universal access to DAA in France’). For each study period, the study population included all adult people residing in metropolitan France and identified as having chronic hepatitis C during that period. The entry date for each period was defined for each individual as the latest date from the following: the start date of the study period, the date the individual's chronic hepatitis C was identified in the SNDS, and the date of the individual's 18th birthday. The exit date for each period was defined for each individual as the earliest date from the following: DAA initiation date (i.e., defined as the date of the first reimbursement for DAA as recorded in the SNDS) and the censoring date (i.e., the study period end date or date of death), whichever occurred first. Of note, individuals identified as having chronic hepatitis C before universal access to DAA contributed to the analyses for both periods, unless DAA treatment had been initiated before August 3, 2017.

### 2.4 Study Outcome

For each study period, the study outcome was time between the period entry date and DAA initiation date. For this outcome, DAA refers exclusively to second‐generation direct‐acting antivirals; first‐generation DAA treatments, including boceprevir‐ and telaprevir‐based regimens, are excluded.

### 2.5 Variables Tested

Along with sex, several socioeconomic variables were tested for their potential association with DAA initiation. These included (i) age, (ii) benefiting from complementary state health insurance for legal residents called CSS (*Complémentaire santé solidaire*), which is free of charge or partially payable depending on an individual's financial means, and (iii) benefiting from a free state medical assistance program called AME (*Aide médicale de l'État*) which guarantees free healthcare access for undocumented residents with limited financial resources. We also tested for prison detention using health insurance data in the SNDS on beneficiaries of France's prisoners' health insurance scheme. Clinical characteristics were also considered in the analyses, including liver disease severity (i.e., severe liver fibrosis, compensated or decompensated cirrhosis, and hepatocellular carcinoma), co‐infection with hepatitis B virus (HBV) or HIV, and chronic renal failure. Current or previous HCV treatment (data available since 2006) was also tested (distinguishing interferon‐based regimens from first‐generation DAA treatment), as were receiving care for alcohol use disorder, receiving care for psychiatric disorders (including mood disorders, psychotic disorders, and other psychiatric disorders) and receiving opioid substitution therapy (OST) (buprenorphine, methadone, naltrexone, or naloxone). These clinical characteristics were identified using standard algorithms developed by the French national health insurance fund [18, 19, 20] (Table S1). For descriptive purposes, a binary variable was created to indicate whether an individual met at least one of the eligibility criteria used for DAA initiation prior to universal access, as recorded in the SNDS. These criteria included HBV infection, HIV infection, alcohol use disorder, receiving OST (as a proxy for injecting drug use), detention, chronic renal failure, and liver disease severity. All variables tested were time‐dependent, with a yearly update.

### 2.6 Statistical Analyses

For each study period, we described the overall socioeconomic and clinical characteristics at the entry date and then compared them according to sex (Chi‐square test). DAA initiation incidence rates per 100 person‐years (PY) and their associated 95% CI were estimated for each study period and were then compared between men and women (exact test for incidence rate difference). For each period, we also described DAA incidence rates and their 95% CI overall and by sex after stratifying individuals based on whether or not they received OST.

For each study period, we used a Cox proportional hazards model to test for potential sex disparities in DAA initiation after adjustment for socioeconomic and clinical characteristics. We first performed univariable analyses to test the different variables; those with a *p*‐value < 0.20 for at least one of the two study periods were considered eligible for the multivariable analyses. A backward variable selection was applied to build the two final multivariable models with variables reaching a significance level of 5% for at least one of the two study periods retained. Population attributable fractions (PAF) were estimated for each variable in these final multivariable models, except for sex and age. PAF are defined as the relative change in the rate of DAA initiation that would be expected to occur in the absence of exposure to a specific factor within the population [21]. In a sensitivity analysis, we estimated both DAA initiation incidence rates and time to DAA initiation, and we replicated Cox models after restricting the study population to individuals newly identified as having chronic hepatitis C (i.e., individuals initiating care for hepatitis C according to the standard algorithm (Table 1)) in each study period. Statistical analyses were performed using STATA software version 19.

## 3 Results

### 3.1 Characteristics of Adults With Chronic Hepatitis C During the Two Study Periods

In study period 1 (i.e., before universal access to DAA), the study population comprised 97 817 individuals, including 59 920 men (61.3%). At the period 1 entry date, the age class most represented in the study population was 50 to 64 years (43.2%), 89.5% of individuals had no previous or current interferon‐based or first‐generation DAA treatment, and 83.3% had no liver disease severity (Table 2).

**TABLE 2:** Characteristics of adult individuals with chronic hepatitis C in metropolitan France at each period entry date, before (i.e., 1 January 2014 to 3 August 2017) and after (4 August 2017 to 31 December 2022) the introduction of universal access to direct‐acting antiviral treatment (ANRS FANTASIO 2 study).

	Before universal access to DAA				After universal access to DAA			
	Overall (*N* = 97 817)	Men (*N* = 59 920)	Women (*N* = 37 897)	*p* ^a^	Overall (*N* = 98 020)	Men (*N* = 60 749)	Women (*N* = 37 271)	*p* ^a^
	Number of individuals (%)				Number of individuals (%)			
Follow‐up time (in months)	21.7 [7.6 – 41.1]	20.5 [7.1–40.6]	24.0 [8.3–41.8]	<* 0.001*	20.8 [3.1–65.0]	23.2 [3.2–65.0]	17.3 [3.0–65.0]	<* 0.001*
Age (in years)				< *0.001*				<* 0.001*
Under 30	4377 (4.5)	3033 (5.1)	1344 (3.5)		4448 (4.5)	3010 (5.0)	1438 (3.9)	
30 to 49	33 601 (34.4)	24 269 (40.5)	9332 (24.6)		28 789 (29.4)	20 688 (34.1)	8101 (21.7)	
50 to 64	42 279 (43.2)	26 265 (43.9)	16 014 (42.3)		41 799 (42.6)	27 020 (44.5)	14 779 (39.7)	
65 to 74	10 899 (11.1)	4165 (7.0)	6734 (17.8)		13 048 (13.3)	6323 (10.4)	6725 (18.0)	
75 and over	6661 (6.8)	2188 (3.7)	4473 (11.8)		9936 (10.1)	3708 (6.1)	6228 (16.7)	
State medical assistance^b^	4233 (4.3)	3273 (5.5)	960 (2.5)	< *0.001*	2854 (2.9)	2024 (3.3)	830 (2.2)	< *0.001*
Complementary state health insurance^c^	19 766 (20.2)	13 404 (22.4)	6362 (16.8)	< *0.001*	23 120 (23.6)	15 318 (25.2)	7802 (20.9)	< *0.001*
Receiving care for alcohol use disorder^d^	9446 (9.7)	7091 (11.8)	2355 (6.2)	< *0.001*	8236 (8.4)	6255 (10.3)	1981 (5.3)	< *0.001*
Receiving opioid substitution therapy^e^	13 942 (14.3)	11 094 (18.5)	2848 (7.5)	<* 0.001*	13 443 (13.7)	10 579 (17.4)	2864 (7.7)	< *0.001*
Prison detention^f^	3849 (3.9)	3677 (6.1)	172 (0.5)	< *0.001*	6845 (7.0)	6429 (10.6)	416 (1.1)	< *0.001*
HBV co‐infection^g^	3254 (3.3)	2315 (3.9)	939 (2.5)	< *0.001*	5383 (5.5)	3766 (6.2)	1617 (4.3)	< *0.001*
HIV co‐infection^g^	11 686 (11.9)	8656 (14.5)	3030 (8.0)	<* 0.001*	9567 (9.8)	7252 (11.9)	2315 (6.2)	< *0.001*
Liver disease severity^g^				< *0.001*				< *0.001*
None	81 516 (83.3)	49 108 (82.0)	32 408 (85.5)		86 752 (88.5)	53 057 (87.3)	33 695 (90.4)	
Fibrosis or compensated cirrhosis	9638 (9.9)	6228 (10.4)	3410 (9.0)		5248 (5.4)	3425 (5.6)	1823 (4.9)	
Decompensated cirrhosis	4517 (4.6)	2967 (5.0)	1550 (4.1)		3565 (3.6)	2367 (3.9)	1198 (3.2)	
Hepatocellular carcinoma	2146 (2.2)	1617 (2.7)	529 (1.4)		2455 (2.5)	1900 (3.1)	555 (1.5)	
Chronic renal failure^g^	2436 (2.5)	1541 (2.6)	895 (2.4)	*0.040*	4936 (5.0)	3221 (5.3)	1715 (4.6)	< *0.001*
Eligible for DAA initiation according to pre‐universal access clinical criteria^h^	44 366 (45.4)	32 182 (53.7)	12 184 (32.2)	<* 0.001*	43 525 (44.4)	32 209 (53.0)	11 316 (30.4)	< *0.001*
Psychiatric disorder^g^, ^i^	26 359 (26.9)	15 321 (25.6)	11 038 (29.1)	< *0.001*	25 145 (25.7)	14 675 (24.2)	10 470 (28.1)	<* 0.001*
Past/current HCV treatment^j^				< *0.001*				<* 0.001*
None	87 505 (89.5)	52 386 (87.4)	35 119 (92.7)		92 237 (94.1)	56 445 (92.9)	35 792 (96.0)	
First‐generation DAA	3574 (3.7)	2446 (4.1)	1128 (3.0)		2072 (2.1)	1416 (2.3)	656 (1.8)	
Interferon‐based therapy	6738 (6.9)	5088 (8.5)	1650 (4.4)		3711 (3.8)	2888 (4.8)	823 (2.2)	

Abbreviations: DAA, direct‐acting antivirals; HBV, hepatitis B virus; HCV, hepatitis C virus.

^a^ Chi‐square test for the comparison of characteristics between men and women.

^b^ In France, state medical assistance is a measure guaranteeing free access to healthcare for persons with irregular immigration status and limited financial resources.

^c^ Complementary state health insurance, free of charge or with a financial contribution, depending on individual's financial resources.

^d^ Individuals receiving care for alcohol use disorder during the current or the previous year were identified using a standard algorithm based on alcohol‐related hospital claims, treatment dispensation, and long‐term disease status during this period (Table S1).

^e^ Individuals receiving opioid substitution therapy during the current or the previous year were identified using a standard algorithm based on dispensation of treatment for opioid substitution and/or opioid overdose (buprenorphine, methadone, naltrexone, naloxone) during this period (Table S1).

^f^ Individuals who had been detained in prison during the current or previous year were identified through enrolment in the prisoners' health insurance scheme.

^g^ Individuals with viral co‐infections, liver disease severity, psychiatric disorders, or chronic renal failure were identified using standard algorithms based on related hospital claims, laboratory tests, treatment dispensation, and long‐term disease status (Table S1).

^h^ Before universal access, eligibility to DAA treatment was based on meeting at least one of the following criteria (available in the SNDS): HBV infection, HIV infection, alcohol use disorder, receiving opioid substitution therapy, prison detention, chronic renal failure, and liver disease severity.

^i^ Psychiatric disorders included mood disorders, psychotic disorders, and other psychiatric disorders.

^j^ Data on the history of HCV treatment were available from 2006. The first‐generation DAA category, including boceprevir‐ and telaprevir‐based regimens, may also include current or previous interferon‐based therapy. Second‐generation DAA treatments (study outcome) are not included.

In study period 2 (i.e., after universal access to DAA), the study population comprised 98 020 individuals, including 60 749 men (62.0%). As for study period 1, the age class most represented at the period 2 entry date was 50–64 years (42.6%). The vast majority of individuals (94.1%) had no previous or current HCV treatment or liver disease severity (88.5%) (Table 2).

For both study periods, there were significant sex disparities in characteristics at the period entry date. Women were less likely to have HBV or HIV co‐infection, liver disease severity, chronic renal failure, previous or current HCV treatment, and less likely to receive OST or to receive care for alcohol use disorder (Table 2). In study period 1, 45.4% of the study population met at least one of the then‐current eligibility criteria for DAA treatment. Specifically, women were less likely to meet one such criterion (32.2% vs. 53.7%, respectively; *p* < 0.001). In study period 2, 44.4% of individuals met at least one of these same pre‐universal access criteria, and women were still less likely to meet at least one of the pre‐universal access criteria (30.4% vs. 53.0%, *p* < 0.001). Moreover, compared with men, lower percentages of women benefited from complementary state health insurance and from state medical assistance. In addition, the percentage of individuals benefiting from France's prisoners' health insurance scheme during the relevant year was also lower among women than in men. By contrast, women accounted for higher percentages of individuals aged over 65 and individuals with psychiatric disorders (Table 2).

### 3.2 Incidence Rates of DAA Initiation During the Two Study Periods

Overall, 51 844 (out of a total of 203 837 PY) and 48 520 (out of a total of 244 305 PY) persons initiated DAA before and after universal access was introduced, respectively, with higher DAA initiation rates before than after universal access (incidence rate [95% CI]: 25.4 [25.2–25.7] and 19.9 [19.7–20.0] per 100 PY, respectively). Specifically, 20 038 women (out of a total of 81 745 PY) and 31 806 men (out of a total of 96 040 PY) initiated DAA before universal access, while 19 656 women (out of a total of 88 326 PY) and 28 864 men (out of a total of 122 092 PY) initiated this treatment after universal access. Before universal access, the incidence rate of DAA initiation was significantly lower in women than in men (24.5 [24.2–24.9] vs. 26.1 [25.8–26.3] per 100 PY, *p* < 0.001). After universal access was introduced, this rate was significantly higher in women than in men (22.3 [21.9–22.6] vs. 18.5 [18.3–18.7] per 100 PY, *p* < 0.001).

### 3.3 Incidence Rates of DAA Initiation According to OST Status

Before universal access, the incidence rate of DAA initiation was lower in individuals who were on OST than in those who were not (20.8 [20.3–21.3] vs. 26.3 [26.0–26.5] per 100 PY, *p* < 0.001). During this study period, the DAA initiation rate was lower in women receiving OST than in their male counterparts (17.8 [16.8–18.9] vs. 21.6 [21.0–22.2] per 100 PY, *p* < 0.001).

After universal access to DAA was introduced, the incidence rate of DAA initiation was higher in individuals receiving OST than in those who were not (26.3 [25.8–26.9] vs. 18.9 [18.7–19.1] per 100 PY, *p* < 0.001); however, we detected no significant sex disparity among individuals receiving OST (25.5 [24.3–26.7] in women vs. 26.6 [25.9–27.2] in men per 100 PY, *p* = 0.116).

### 3.4 Sex Disparities in DAA Initiation

Table 3 presents univariable and multivariable analyses of associations with DAA initiation, before and after universal access to DAA was introduced in France. Before universal access, DAA initiation was significantly lower in women than in men (adjusted hazard ratio (aHR) [95% CI]: 0.91 [0.90–0.93], *p* < 0.001) after adjustment for age, socioeconomic variables, prison detention, liver disease severity, comorbidities, previous or current HCV treatment, receiving OST, and care for alcohol use disorder. After universal access, DAA initiation was significantly higher in women than in men (1.08 [1.06–1.10], *p* < 0.001) after multivariable adjustment for the same variables.

**TABLE 3:** Sex disparities in direct‐acting antiviral initiation among adult individuals with chronic hepatitis C in metropolitan France, before (i.e., 1 January 2014 to 3 August 2017) and after (4 August 2017 to 31 December 2022) the introduction of universal access to this treatment, univariable and multivariable analyses, Cox proportional hazards models (ANRS FANTASIO 2 study).

	Univariable analyses				Multivariable analyses					
	Before universal access to DAA		After universal access to DAA		Before universal access to DAA			After universal access to DAA		
	HR [95% CI]	*p*	HR [95% CI]	*p*	aHR [95% CI]	*p*	PAF (%)	aHR [95% CI]	*p*	PAF (%)
Female sex	0.95 [0.94–0.97]	*< 0.001*	1.14 [1.12–1.16]	*< 0.001*	0.91 [0.90–0.93]	*< 0.001*		1.08 [1.06–1.10]	*< 0.001*	
Age (in years)										
Under 30	0.34 [0.32–0.36]	*< 0.001*	0.51 [0.49–0.54]	*< 0.001*	0.36 [0.34–0.39]	*< 0.001*		0.50 [0.48–0.53]	*< 0.001*	
30 to 49	0.64 [0.63–0.65]	*< 0.001*	0.82 [0.80–0.83]	*< 0.001*	0.67 [0.65–0.68]	*< 0.001*		0.80 [0.78–0.82]	*< 0.001*	
50 to 64 (ref.)	1		1		1			1		
65 to 74	1.01 [0.98–1.04]	0.434	0.85 [0.82–0.87]	*< 0.001*	1.06 [1.03–1.08]	*< 0.001*		0.91 [0.88–0.94]	*< 0.001*	
75 and over	0.84 [0.81–0.87]	*< 0.001*	0.60 [0.58–0.63]	*< 0.001*	0.87 [0.83–0.90]	*< 0.001*		0.62 [0.60–0.65]	*< 0.001*	
State medical assistance^a^	0.94 [0.89–0.99]	0.015	1.32 [1.26–1.38]	*< 0.001*	1.32 [1.25–1.39]	*< 0.001*	0.8	1.70 [1.62–1.78]	*< 0.001*	1.8
Complementary state health insurance^b^	0.87 [0.85–0.89]	*< 0.001*	1.24 [1.22–1.27]	*< 0.001*	1.02 [0.99–1.04]	0.128	0.3	1.23 [1.20–1.25]	*< 0.001*	4.7
Receiving care for alcohol use disorder^c^	1.03 [1.00–1.06]	0.023	1.16 [1.13–1.20]	*< 0.001*	0.94 [0.91–0.97]	*< 0.001*	−0.6	0.99 [0.95–1.02]	*0.402*	−0.1
Receiving opioid substitution therapy^d^	0.80 [0.78–0.82]	*< 0.001*	1.26 [1.24–1.29]	*< 0.001*	0.88 [0.85–0.90]	*< 0.001*	−1.7	1.18 [1.15–1.21]	*< 0.001*	2.5
Prison detention^e^	0.71 [0.68–0.75]	*< 0.001*	0.80 [0.77–0.83]	*< 0.001*	0.94 [0.89–0.99]	0.017	−0.2	0.70 [0.67–0.73]	*< 0.001*	−3.0
HBV co‐infection^f^	1.01 [0.96–1.06]	0.627	0.45 [0.43–0.48]	*< 0.001*	0.81 [0.77–0.86]	*< 0.001*	−0.8	0.45 [0.43–0.48]	*< 0.001*	−3.3
HIV co‐infection^f^	1.55 [1.52–1.59]	*< 0.001*	0.77 [0.74–0.79]	*< 0.001*	1.61 [1.57–1.65]	*< 0.001*	5.8	0.81 [0.78–0.84]	*< 0.001*	−1.9
Liver disease severity										
None (ref.)			1		1			1		
Fibrosis or compensated cirrhosis	1.75 [1.70–1.80]	*< 0.001*	1.04 [1.00–1.08]	*0.057*	1.69 [1.65–1.74]	*< 0.001*	5.3	1.01 [0.97–1.05]	*0.647*	0.1
Decompensated cirrhosis	2.21 [2.14–2.30]	*< 0.001*	1.31 [1.25–1.37]	*< 0.001*	2.16 [2.08–2.24]	*< 0.001*	3.5	1.31 [1.25–1.38]	*< 0.001*	0.9
Hepatocellular carcinoma	2.00 [1.90–2.11]	*< 0.001*	1.15 [1.07–1.22]	*< 0.001*	1.79 [1.70–1.89]	*< 0.001*	1.3	1.12 [1.05–1.20]	*0.001*	0.2
Psychiatric disorders^f^, ^g^	0.94 [0.92–0.96]	*< 0.001*	1.07 [1.05–1.10]	*< 0.001*	0.93 [0.91–0.95]	*< 0.001*	−2.0	0.98 [0.96–1.00]	*0.031*	−0.6
Chronic renal failure^f^	0.62 [0.58–0.66]	*< 0.001*	0.18 [0.17–0.20]	*< 0.001*	0.56 [0.53–0.60]	*< 0.001*	−1.3	0.18 [0.16–0.19]	*< 0.001*	−5.1
Past/current HCV treatment^h^										
None (ref.)	1		1		1			1		
First‐generation DAA	0.59 [0.56–0.62]	*< 0.001*	0.11 [0.09–0.13]	*< 0.001*	0.53 [0.50–0.56]	*< 0.001*	−2.5	0.10 [0.08–0.11]	*< 0.001*	−3.3
Interferon‐based therapy	0.78 [0.75–0.81]	*< 0.001*	0.11 [0.10–0.12]	*< 0.001*	0.78 [0.75–0.81]	*< 0.001*	−1.8	0.10 [0.09–0.11]	*< 0.001*	−5.7

Abbreviations: (a)HR, (adjusted) hazard ratio; CI, confidence interval; DAA, direct‐acting antiviral; HBV, hepatitis B virus; HCV, hepatitis C virus; PAF, population attributable fractions; ref., reference.

^a^ In France, state medical assistance is a measure guaranteeing free access to healthcare for persons with irregular immigration status and limited financial resources.

^b^ State complementary health insurance, free of charge or with a financial contribution, depending on individuals' financial resources.

^c^ Individuals receiving care for alcohol use disorders during the current or the previous year were identified using a standard algorithm based on alcohol‐related hospital claims, treatment dispensation, and long‐term disease status during this period (Table S1).

^d^ Individuals receiving opioid substitution therapy during the current or the previous year were identified using a standard algorithm based on dispensation of treatment for opioid substitution and/or opioid overdose (buprenorphine, methadone, naltrexone, naloxone) during this period (Table S1).

^e^ Individuals who had been detained in prison during the current or previous year were identified through enrollment in the prisoners' health insurance scheme.

^f^ Individuals with viral co‐infections, liver disease severity, psychiatric disorders, or chronic renal failure were identified using standard algorithms based on related hospital claims, laboratory tests, treatment dispensation, and long‐term disease status (Table S1).

^g^ Psychiatric disorders included mood disorders, psychotic disorders, and other psychiatric disorders.

^h^ Data on the history of HCV treatment were available from 2006. The first‐generation DAA category, including boceprevir‐ and telaprevir‐based regimens, may also include current or previous interferon‐based therapy. Second‐generation DAA treatments (study outcome) are not included.

### 3.5 Other Disparities in DAA Initiation

For both study periods, receiving state medical assistance, benefiting from state complementary health insurance, and liver disease severity were all positively associated with DAA initiation in multivariable analyses, while belonging to the youngest (18–49 years) and oldest age classes (75 years and over), HBV co‐infection, psychiatric disorders, chronic renal failure, and previous or current HCV treatment were all negatively associated with DAA initiation. Receiving OST was negatively associated with DAA initiation before universal access, but positively associated after its introduction; the reverse was observed for HIV co‐infection. Receiving care for alcohol use disorder was negatively associated with DAA initiation only before universal access was introduced. Prison detention was negatively associated with DAA initiation before and after it was introduced (Table 3).

Before universal access, the highest positive PAF values were obtained for HIV co‐infection and liver disease severity. After universal access was introduced, receiving state complementary health insurance had the highest positive PAF value, while chronic renal failure and previous or current interferon‐based or first‐generation DAA therapy had the highest negative PAF values.

### 3.6 Sensitivity Analysis

When the analysis was restricted to individuals newly identified as having chronic hepatitis C, who accounted for 52.2% and 48.3% of the study population in the first and second study period, respectively, the same significant sex differences in characteristics at study period entry date were observed as in the overall study population (Table S2).

In this population, time to DAA initiation was shorter after universal access to DAA than before (median [IQR]: 1.9 [0.5–4.2] vs. 4.2 [1.6–9.7] months).

Before universal access, the incidence rate of DAA initiation was not significantly different between women and men (36.0 [35.3–36.7] vs. 36.2 [35.6–36.7] per 100 PY, *p* = 0.692). After universal access was introduced, this rate was significantly higher in women than in men (53.2 [52.2–51.3] vs. 46.8 [46.1–47.5] per 100 PY, *p* < 0.001). In the multivariable Cox model adjusted for age, socioeconomic variables, prison detention, liver disease severity, comorbidities, previous or current HCV treatment, receipt of OST, and care for alcohol use disorder, DAA initiation was significantly lower in women than in men, both before (aHR [95% CI]: 0.95 [0.92–0.97], *p* < 0.001) and after universal access (0.96 [0.93–0.98], *p* < 0.001) (Table S3).

## 4 Discussion

In France, making DAA universally available to all persons with chronic hepatitis C has helped to increase women's uptake of this essential treatment. Before this policy, men were more likely to initiate DAA, largely because of sex discrepancies in risk factors for disease progression. This study is the first to clearly show that universal access to DAA has contributed to closing this gap in the French context, and that it even reversed the previously observed sex difference in treatment initiation.

More specifically, our results highlight a change in sex disparities in DAA initiation over time. Analysis of data recorded before universal access showed a lower incidence rate in women than in men, while the reverse was found from data recorded after universal access. These sex disparities in DAA initiation were confirmed after multivariable adjustment for socioeconomic characteristics, liver disease severity, comorbidities, prison detention, previous or current HCV treatment (i.e., first‐generation DAA or interferon‐based therapies), as well as receiving care for alcohol use disorder and receiving OST.

Universal access is part of a set of measures included in the French national health policy in 2017–2019 targeting HCV elimination by 2025. Two of these measures, aimed at simplifying access to treatment, include dispensing DAA in community pharmacies, which was introduced in 2018 (prior to this, only hospitals could dispense DAA), and the expansion of prescription rights to all physicians for patients without severe liver disease, comorbidities, or previous or current HCV treatment, which was introduced in 2019 (prior to this, only HCV specialists working in hospital services could prescribe DAA) [8]. Although France did not achieve its goal for HCV elimination by 2025, this set of measures has helped put the country on track towards meeting the WHO's elimination targets by 2030. In 2020, a modelling study evaluated progress towards HCV elimination in 45 high‐income countries, using 2017 screening and treatment rates as the baseline [22]. Only nine countries—France, Australia, Iceland, Italy, Japan, South Korea, Spain, Switzerland, and the United Kingdom—were found to be on track for elimination by 2030. Many countries have now removed clinical eligibility criteria for HCV treatment initiation. Among the 33 countries and territories in the 2024 global report on national policies, programs, and progress towards HCV elimination [23] (note that France was not included in this report), 31 had removed fibrosis stage requirements as a condition for initiating HCV treatment. However, treatment was free of charge for patients in only 17 (52%) of these countries and territories, while co‐payment exoneration was partially adopted in 10 other countries and territories. In France, DAA treatment is fully reimbursed by the National Health Insurance system for all individuals with chronic hepatitis C benefiting from social security coverage [24]. This has certainly contributed to improving equity in access to care, even though socioeconomic disparities persist regarding HCV‐related morbidity and mortality [25]. Our study complements Pol et al.'s work, which analysed trends in HCV screening and time to HCV treatment initiation in France between 2015 and 2019 [10]. In our study, when analyses were restricted to individuals newly identified as having chronic hepatitis C in the SNDS during the study period, we found a shorter time to DAA initiation after universal access was introduced. This confirms that the extension of DAA availability to all persons with chronic hepatitis C in the country and the elimination of multidisciplinary team meetings to discuss DAA initiation for patients without severe liver disease, comorbidities, or previous or current HCV treatment have contributed to substantially shortening delays in treatment access.

Importantly, for both periods in our study, analyses showed sex disparities in the clinical characteristics at the entry date, with women having lower percentages of comorbidities—including HIV co‐infection—and of liver disease severity, compared with men [26]. These were the primary reasons for DAA initiation before universal access to DAA, as confirmed by our estimated PAF, whose values were highest for HIV co‐infection and liver disease severity in the multivariable model for this period. Therefore, the lower incidence rate of DAA initiation observed in women (compared with men) before universal access is mostly explained by sex disparities in clinical characteristics.

Of note, the percentage of psychiatric disorders at entry date was higher among women than among men for both periods, reflecting the fact that mood disorders were more frequent in women than in men in the two periods (data not shown); these findings align with observed sex disparities in the general populations of European countries, in particular for internalized psychiatric disorders (e.g., anxiety and depression) [27]. By contrast, at the entry date for both periods, the percentage of women benefiting from social assistance measures for healthcare access, as well as the percentage of women detained in prison, were both lower than those of men, confirming trends observed for the French general population [28, 29].

Our study shows lower incidence rates of DAA initiation in France after universal access was introduced. These findings align with French epidemiological data indicating a marked decline in treatment initiation in the years after the introduction of universal access; this reflects both the impact of the COVID‐19 pandemic and the shrinking pool of individuals remaining to be treated [8].

Our study also confirms that disparities in DAA initiation persist for individuals with psychiatric disorders, a population with less treatment coverage than the other groups living with chronic hepatitis C, even after the introduction of universal access to DAA [10]. This population accounted for more than a quarter of our study sample. A combination of factors may explain this disparity, including financial constraints for psychiatric hospitals [30] and the need for close monitoring to prevent non‐adherence to treatment and to avoid drug interactions [10]. In the context of the ongoing effort to eliminate HCV in France, it is essential to overcome these barriers in order to ensure that the greatest number of individuals with psychiatric disorders who are living with chronic hepatitis C have access to DAA. Along with benefits at the individual and public health levels, overcoming these barriers could also result in cost savings for the national health system, as shown by the lowered frequency and duration of hospitalizations observed in the psychiatric population after HCV cure [31].

Another interesting finding of our study is that receiving OST was positively associated with DAA initiation after universal access was introduced, whereas it was negatively associated beforehand. In addition, we detected no significant sex disparities in DAA initiation among individuals receiving OST after universal access was introduced, unlike pre‐universal data in the early DAA era which showed lower DAA initiation rates among women receiving OST compared with men [11]. These encouraging results can be considered within the wider picture of increased comprehensive efforts in France to improve the HCV cascade of care among people who inject drugs, specifically through the development of outreach and point‐of‐care services [32, 33], integrated multidisciplinary care and test‐and‐treat initiatives [32], as well as peer‐led interventions such as AERLI (*Accompagnement et Education aux Risques Liés à l'Injection*) to educate people on safer injection practices, to raise HCV testing uptake, and to reduce risk behaviors [34]. Studies providing a more detailed understanding of the profiles and needs of men and women who test positive for HCV in harm reduction facilities may also have contributed to improve access to DAA in this population [35].

Of note, in our study, HBV co‐infection was negatively associated with DAA initiation both before and after universal access to DAA was introduced. This may be partially explained by the need for an appropriate evaluation and management of the risk of HBV reactivation in patients receiving DAA [36].

Finally, our results highlight that benefiting from state medical assistance or state complementary health insurance facilitates DAA initiation; this is true for both before and after the introduction of universal access and underlines the positive impact of these social programs on improving access to care for socially vulnerable subgroups of individuals with chronic HCV infection.

### 4.1 Analysis Restricted to Individuals Newly Identified as Having Chronic Hepatitis C

Interestingly, results of the sensitivity analysis showed higher incidence rates of DAA initiation in both study periods, before and after universal access, than those observed in the main analysis. This suggests that individuals newly identified as having chronic hepatitis C may be more likely to enter an active care pathway, with treatment initiation appearing to occur relatively soon after their diagnosis, as indicated by estimates of time to treatment initiation, particularly after the introduction of universal access.

Importantly, the sensitivity analysis confirmed a higher incidence rate of DAA initiation in women than in men after the introduction of universal access. The negative association between female sex and DAA initiation observed in the multivariable Cox model may be explained by an interaction between sex and prison detention (data not shown), with women having a higher hazard of DAA initiation than men among individuals with a history of incarceration.

### 4.2 Study Strengths and Limitations

The use of the French National Health Data System database, which covers almost all of the French general population, and the 9‐year duration of the whole study period, spanning both pre‐ and post‐universal DAA access, are two major strengths of our study. However, two important limitations must be acknowledged, and both are associated with the content of the SNDS, which is primarily structured as a medico‐administrative database. First, the SNDS does not include biomedical information, such as blood test results or data from liver fibrosis staging tests. In addition, socioeconomic information available in the database is limited, and no behavioural data are recorded; accordingly, it is difficult to identify certain comorbidities, such as at‐risk alcohol consumption. Indeed, we used receiving care for alcohol use disorder as a proxy for this. In addition, we were unable to analyse in detail the specific sex‐related barriers (e.g., desire for pregnancy, family responsibilities, etc.) that could explain the sex differences in the initiation of DAA. Second, only individuals who received healthcare reimbursements for chronic hepatitis C care are present in the database; this means that individuals diagnosed with chronic hepatitis C who did not subsequently engage with the healthcare system were not represented in our analyses. In the absence of mandatory HCV notification and associated registry in France, these individuals could not be identified.

## 5 Conclusions

In France, universal access to DAA has reversed sex disparities in DAA initiation among people with chronic hepatitis C. This change likely reflects differences in disease progression profiles between men and women. To ensure truly equitable access to HCV care, current research should explore whether reversal or reduction of sex disparities is observed in other countries and subpopulations.

## Author Contributions

Conceptualization: F.M., P.C., B.R., C.P.; methodology: F.M., C.B., C.P.; software: C.R., V.D.B.; validation: F.M., C.R., V.D.B., C.P.; statistical analysis: C.R., V.D.B., CP; interpretation of results: all authors; data curation: F.M., C.R., V.D.B., C.P.; writing – original draft: F.M., C.R.; writing – review and editing: all authors; supervision: F.M., C.P.; project administration: F.M.; funding acquisition: F.M., P.C., C.P. All authors reviewed and approved the final version of the submitted manuscript.

## Funding

The FANTASIO 2 research project was funded by the French National Agency for Research on HIV/AIDS, viral hepatitis and emerging infectious diseases (ANRS‐MIE).

## Ethics Statement

Since 2021, research teams within the French National Institute for Health and Medical Research (Inserm) have benefited from an expanded permanent access to individual‐level data from the SNDS, under the regulatory framework established by the decree of 30 June 2021. This access remains subject to individual habilitation procedures and is restricted to research activities falling within Inserm's public service missions. The authors of the present study who performed the analyses were accredited to SNDS database access.

## Conflicts of Interest

Marc Bourliere declares collaborations with Gilead, AbbVie, GSK, Janssen, and Precision BioSciences. Philippe Sogni and Benjamin Rolland declare collaborations with Gilead and AbbVie. The other authors declare no conflict of interest with regard to the content of the manuscript.

## Supporting information


**Table S1:** Algorithms used for the identification of liver disease severity and comorbidities (ANRS FANTASIO 2 study).
**Table S2:** Characteristics of adult individuals newly identified as having chronic hepatitis C in metropolitan France at each period entry date, before (i.e., 1 January 2014 to 3 August 2017) and after (4 August 2017 to 31 December 2022) the introduction of universal access to direct‐acting antiviral treatment (ANRS FANTASIO 2 study).
**Table S3:** Sex disparities in direct‐acting antiviral initiation among adult individuals newly identified as having chronic hepatitis C in metropolitan France, before (i.e., 1 January 2014 to 3 August 2017) and after (4 August 2017 to 31 December 2022) the introduction of universal access to this treatment, univariable and multivariable analyses, Cox proportional hazards models (ANRS FANTASIO 2 study).

## Data Availability

The dataset analysed in this study was derived from the French National Health Data System (SNDS). Owing to legal and regulatory requirements, SNDS data cannot be freely accessed and are available only to health insurance organizations and a restricted number of authorized French public institutions.
