# Supplementary material for: Sex Disparities in Hepatitis C Treatment Initiation: Role of Universal Access to DAA in France (ANRS FANTASIO 2)
Source: Liver Int. 2026 Sep 18;46(10):e70884. doi: 10.1111/liv.70884 (PMC13587266; doi:10.1111/liv.70884)
Supplement: Supplementary file 1 [file LIV-46-0-s001.docx]

**Supplementary Table 1 -** **Algorithms used for the identification of liver disease severity and comorbidities (ANRS FANTASIO 2 study)**

| **Comorbidities** | **Algorithm** | **Drug ATC codes** |
| --- | --- | --- |
| HBV infection^18^ | **At least one of the following criteria during year N**:   - Active or incident LTD for hepatitis B (ICD-10 codes B18.0 and B18.1) - Hospital claims (main, related or associated diagnosis) for hepatitis B (ICD-10 codes B18.0 and B18.1) - ≥3 laboratory diagnostic tests for HBV DNA detection and quantification (NABM 4120) - Dispensing of HBV treatment at ≥3 different dates | (Adefovir dipivoxil) J05AF08; (entecavir) J05AF10; (telbuvidine) J05AF11; (lamivudine) J05AF05; (tenofovir disoproxil) J05AF07 |
| HIV infection^20^ | **At least one of the following criteria during year N:**   - Active or incident LTD for HIV (ICD-10 codes B20-B24, F02.4, and Z21) - Hospital claims (main, related or associated diagnosis) for HIV (ICD-10 codes B20-B24, F02.4, and Z21) - ≥1 laboratory tests related to HIV (NABM 805, 806, 1691, 4117, 4122) - Dispensing of HIV treatment at ≥3 different dates without dispensing of HBV treatment on the same dates   **OR (during the years N-4 to N):**   - Hospital claims (main, related or associated diagnosis) for HIV (ICD-10 codes B20-B24, F02.4 and Z21) | (zidovudine) J05AF01, J05AR01, J05AR04; (didanosine) J05AF02; (zalcitabine) J05AF03; (stavudine) J05AF04, J05AR28; (abacavir) J05AF06, J05AR02, J05AR13; (nevirapine) J05AG01, J05AR07, J05AR05; (efavirenz) J05AG03, J05AR06, J05AR11; (etravirine) J05AG04; (rilpivirine) J05AG05, J05AR08, J05AR19, J05AR21; (saquinavir) J05AE01; (indinavir) J05AE02; (ritonavir) J05AR23, J05AR26, J05AR10, J05AE30, J05AE03; (nelfinavir) J05AE04; (amprenavir) J05AE05, J05AE07; (atazanavir) J05AE08, J05AR15; (tipranavir) J05AE09; (darunavir) J05AE10, J05AR14, J05AR22; (emtricitabine) J05AF09, J05AR17, J05AR20, J05AR18, J05AR09, J05AR03; (enfuvirtide) J05AX07; (maraviroc) J05AX09; (cabotegravir) J05AJ04; (doravirine) J05AG06; (dolutegravir) J05AJ03 |
| Severe liver fibrosis or compensated cirrhosis^19^ | - Hospital claims (main, related or associated diagnosis) for severe liver fibrosis or compensated cirrhosis (ICD-10 codes K70.0, K70.1, K70.2, K70.3, K74, and K71.7) during the year N or N-1 and/or - Active or incident LTD for severe liver fibrosis or compensated cirrhosis (ICD-10 codes K70.0, K70.1, K70.2, K70.3, K74 and K71.7) during the year N |  |
| Decompensated cirrhosis^19^ | - Hospital claims (main, related or associated diagnosis) for decompensated cirrhosis (ICD-10 codes R18, I86.4, K76.7, K76.6, I85, and K72) during the year N or N-1 and/or - Active or incident LTD for decompensated cirrhosis (ICD-10 codes R18, I86.4, K76.7, K76.6, I85 and K72) during the year N |  |
| Hepatocellular carcinoma^19^ | Hospital claims (main, related or associated diagnosis) for hepatocellular carcinoma (ICD-10 codes C22.0) during year N or N-1 |  |
| Mood disorders^31^ | **At least one of the following criteria during year N:**   - Active or incident LTD for mood disorders (ICD-10 codes F32-F34, F38-F45 and F48) - Hospital claims (main, related or associated diagnosis) for mood disorders (ICD-10 codes F32-F34, F38-F45 and F48) - Dispensing of treatment for mood disorders | (antidepressants) N06A |
| Psychotic disorders^31^ | **At least one of the following criteria during year N:**   - Active or incident LTD for psychotic disorders (ICD-10 codes F20-F23, F25 and F28-F31) - Hospital claims (main, related or associated diagnosis) for psychotic disorders (ICD-10 codes F20-F23, F25 and F28-F31) - Dispensing of treatment for psychotic disorders | (antipsychotics) N05A; (valpromides) N03AG02; (valproic acid (depakote only)) N03AG01 and CIP 3487623, 3487669, 3544427 |
| Other psychiatric disorders^31^ | **At least one of the following criteria during year N:**   - Active or incident LTD for other psychiatric disorders (ICD-10 codes F04-F07, F09, F50, F52-F54, F59-F66, F68-F73, F78-F84, F88-F95 and F98-F99) - Hospital claims (main, related or associated diagnosis) for other psychiatric disorders (ICD-10 codes F04-F07, F08, F50, F52-F54, F59-F65, F68-F73, F78-F84, F88-F95 and F99) |  |
| Chronic renal failure^20^ | **At least one of the following criteria during year N:**   - Hospital claims for renal transplant (HPG code 27C06) - Reimbursement of medical procedures related to kidney transplants (CCMA codes JAEA003 and HNEA002) - Dispensing of immunosuppressant treatment at ≥3 different dates **AND**   - Hospital claims for renal transplant follow-up (HPG code 11M17) during the years N-4 to N and/or   - Hospital claims (main, related or associated diagnosis) for renal transplant (ICD-10 code Z94.0) during the years N-4 to N and/or   - Hospital claims for renal transplant (HPG code 27C06) during the years N-4 to N and/or   - Reimbursement of medical procedures related to kidney transplants (CCMA codes JAEA003 and HNEA002) during the years N-4 to N and/or   - Active or incident LTD for chronic renal failure (ICD-10 code N18) - Dispensing of ≥45 days of haemodialysis treatment (CCMA codes JVJF004, JVJF008, JVRP004, HPG codes 28Z03Z, 28Z04Z, procedure code D01-D06, D09-D14, D17, D24) - Dispensing of ≥1 day of peritoneal dialysis treatment (CCMA codes JVJB001, JVRP007, JVRP008, YYYY007, HPG codes 28Z01Z, 28Z02Z, procedure code D07, D08, D15, D16, D18-D23) - Dispensing of ≥1 day of haemodialysis treatment (CCMA codes JVJF004, JVJF008, JVRP004, HPG codes 28Z03Z, 28Z04Z, procedure code D01-D06, D09-D14, D17, D24) **AND**   - Dispensing of ≥45 days of haemodialysis treatment (CCMA codes JVJF004, JVJF008, JVRP004, HPG codes 28Z03Z, 28Z04Z, procedure code D01-D06, D09-D14, D17, D24) during the year N-1 and/or   - Dispensing of ≥1 day of peritoneal dialysis treatment (CCMA codes JVJB001, JVRP007, JVRP008, YYYY007, HPG codes 28Z01Z, 28Z02Z, procedure code D07, D08, D15, D16, D18-D23) during year N-1 | (mycophenolic acid) L04AA06; (sirolimus) L04AA10; (everolimus) L04AA18; (ciclosporin) L04AD01; (tacrolimus) L04AD02; (azathioprine) L04AX01 |
| Alcohol use disorder^19^ | **At least one of the following criteria during the years N-1 to N:**   - Hospital claims (main, related or associated diagnosis) for alcohol use disorder (ICD-10 codes K70, K86.0, F10, Z50.2 and Y91) - Dispensing of treatment for alcohol use disorder   **OR:**  Active or incident LTD for alcohol use disorder during year N (ICD-10 codes K70, K86.0, F10, Z50.2 and Y91) | (disulfiram) N07BB01; (acamprosate) N07BB03; (naltrexone) N07BB04; (nalmefene) N07BB05; (baclofen) M03BX01; (other) N07BB02 |
| Opioid substitution therapy^10^ | Dispensing of opioid substitution treatment or opioid overdose treatment during the year N-1 or N | (buprenorphine) N07BC01, N07BC51; (methadone) N07BC02; (naltrexone) N07BB04; (naloxone) V03AB15 |
| Prison detention^10^ | Individuals covered by France’s prison detention health insurance scheme (codes 652, 655, 656, 657, 658, and 659) during the year N. |  |
| State complementary health insurance* | Individuals covered by France’s free complementary health insurance scheme (codes 89, 91, 92, and 93) during the year N |  |
| State medical assistance^10^ | Individuals covered by France’s free state medical assistance health insurance scheme (codes 95, 96, and 652) during the year N. |  |

ATC, Anatomical Therapeutic Classification; CCMA, Common Classification of Medical Acts; HPG, homogeneous patient group; ICD, International Classification of Diseases; LTD, long-term disease status (an administrative measure which provides free treatment and care for people with certain chronic diseases, including hepatitis)

* <https://documentation-snds.health-data-hub.fr/snds/fiches/acs.html#effectifs-acs>

**Supplementary Table 2 - Characteristics of adult individuals newly identified as having chronic hepatitis C in metropolitan France at each period entry date, before (i.e., 1 January 2014 to 3 August 2017) and after (4 August 2017 to 31 December 2022) the introduction of universal access to direct-acting antiviral treatment (ANRS FANTASIO 2 study)**

|  | **Before universal access to DAA** | | | | **After universal access to DAA** | | | | |  |
| --- | --- | --- | --- | --- | --- | --- | --- | --- | --- | --- |
|  | **Overall**  (N = 51,019) | **Men**  (N = 31,910) | **Women**  (N = 19,109) |  | | **Overall**  (N = 47,322) | **Men**  (N = 29,899) | **Women**  (N = 17,423) |  | |
|  | Number of individuals (%) | | | *P-value^1^* | | Number of individuals (%) | | | *P-value^1^* | |
| **Follow-up time** (in months) | 12.5 [3.7 – 26.8] | 12.2 [3.7 – 26.4] | 12.9 [3.6 – 27.3] | *<0.001* | | 4.5 [1.2 – 25.4] | 4.6 [1.2 – 27.8] | 4.4 [1.3 – 22.7] | *<0.001* | |
| **Age** (in years) |  |  |  | *<0.001* | |  |  |  | *<0.001* | |
| - under 30 | 3,348 (6.6) | 2,423 (7.6) | 925 (4.8) |  | | 3,682 (7.8) | 2,510 (8.4) | 1,172 (6.7) |  | |
| - 30 to 49 | 18,032 (35.3) | 13,102 (41.1) | 4,930 (25.8) |  | | 15,509 (32.8) | 11,232 (37.6) | 4,277 (24.5) |  | |
| - 50 to 64 | 20,644 (40.5) | 12,910 (40.5) | 7,734 (40.5) |  | | 18,792 (39.7) | 12,036 (40.3) | 6,756 (38.8) |  | |
| - 65 to 74 | 5,162 (10.1) | 2,126 (6.7) | 3,036 (15.9) |  | | 5,590 (11.8) | 2,666 (8.9) | 2,924 (16.8) |  | |
| - 75 and over | 3,833 (7.5) | 1,349 (4.2) | 2,484 (13.0) |  | | 3,749 (7.9) | 1,455 (4.9) | 2,294 (13.2) |  | |
| **State medical assistance^2^** | 3,531 (6.9) | 2,718 (8.5) | 813 (4.3) | *<0.001* | | 2,547 (5.4) | 1,802 (6.0) | 745 (4.3) | *<0.001* | |
| **Complementary state health insurance^3^** | 12,070 (23.7) | 8,273 (25.9) | 3,797 (19.9) | *<0.001* | | 12,543 (26.5) | 8,527 (28.5) | 4,016 (23.0) | *<0.001* | |
| **Receiving care for alcohol use disorder^4^** | 5,004 (9.8) | 3,753 (11.8) | 1,251 (6.5) | *<0.001* | | 4,133 (8.7) | 3,181 (10.6) | 952 (5.5) | *<0.001* | |
| **Receiving opioid substitution therapy^5^** | 7,067 (13.9) | 5,600 (17.5) | 1,467 (7.7) | *<0.001* | | 6,502 (13.7) | 5,147 (17.2) | 1,355 (7.8) | *<0.001* | |
| **Prison detention^6^** | 3,227 (6.3) | 3,084 (9.7) | 143 (0.7) | *<0.001* | | 4,610 (9.7) | 4,348 (14.5) | 262 (1.5) | *<0.001* | |
| **HBV co-infection^7^** | 2,217 (4.3) | 1,572 (4.9) | 645 (3.4) | *<0.001* | | 2,127 (4.5) | 1,485 (5.0) | 642 (3.7) | *<0.001* | |
| **HIV co-infection^7^** | 6,805 (13.3) | 5,066 (15.9) | 1,739 (9.1) | *<0.001* | | 4,326 (9.1) | 3,411 (11.4) | 915 (5.3) | *<0.001* | |
| **Liver disease severity^7^** |  |  |  | *<0.001* | |  |  |  | *<0.001* | |
| None | 43,160 (84.6) | 26,684 (83.6) | 16,476 (86.2) |  | | 42,210 (89.2) | 26,328 (88.1) | 15,882 (91.2) |  | |
| Fibrosis or compensated cirrhosis | 4,394 (8.6) | 2,848 (8.9) | 1546 (8.1) |  | | 2,103 (4.4) | 1,385 (4.6) | 718 (4.1) |  | |
| Decompensated cirrhosis | 2,436 (4.8) | 1,596 (5.0) | 840 (4.4) |  | | 1,924 (4.1) | 1,301 (4.4) | 623 (3.6) |  | |
| Hepatocellular carcinoma | 1,029 (2.0) | 782 (2.5) | 247 (1.3) |  | | 1,085 (2.3) | 885 (3.0) | 200 (1.1) |  | |
| **Chronic renal failure^7^** | 1,551 (3.0) | 973 (3.0) | 578 (3.0) | *0.876* | | 2,894 (6.1) | 1,901 (6.4) | 993 (5.7) | *0.003* | |
| **Eligible for DAA initiation**  **according to pre-universal access clinical criteria**^8^ | 24,407 (47.8) | 17,945 (56.2) | 6,462 (33.8) | *<0.001* | | 22,210 (46.9) | 16,978 (56.8) | 5,232 (30.0) | *<0.001* | |
| **Psychiatric disorders^7,9^** | 12,758 (25.0) | 7,492 (23.5) | 5,266 (27.6) | *<0.001* | | 11,205 (23.7) | 6,698 (22.4) | 4,507 (25.9) | *<0.001* | |

DAA, direct-acting antivirals; HBV, hepatitis B virus; HCV, hepatitis C virus.

^1^ Chi-square test for the comparison of characteristics between men and women.

^2^ In France, state medical assistance is a measure guaranteeing free access to healthcare for persons with irregular immigration status and limited financial resources.

^3^ Complementary state health insurance, free of charge or with a financial contribution, depending on individual’s financial resources.

^4^ Individuals receiving care for alcohol use disorder during the current or the previous year were identified using a standard algorithm based on alcohol-related hospital claims, treatment dispensation, and long-term disease status during this period (Supplementary Table 1).

^5^ Individuals receiving opioid substitution therapy during the current or the previous year were identified using a standard algorithm based on dispensation of treatment for opioid substitution and/or opioid overdose (buprenorphine, methadone, naltrexone, naloxone) during this period (Supplementary Table 1).

^6^ Individuals who had been detained in prison during the current or previous year were identified through enrolment in the prisoners’ health insurance scheme.

^7^ Individuals with viral co-infections, liver disease severity, psychiatric disorders, or chronic renal failure were identified using standard algorithms based on related hospital claims, laboratory tests, treatment dispensation, and long-term disease status (Supplementary Table 1).

^8^ Before universal access, eligibility to DAA treatment was based on meeting at least one of the following criteria (available in the SNDS): HBV infection, HIV infection, alcohol use disorder, receiving opioid substitution therapy, prison detention, chronic renal failure, and liver disease severity.

^9^ Psychiatric disorders included mood disorders, psychotic disorders, and other psychiatric disorders.

**Supplementary Table 3 - Sex disparities in direct-acting antiviral initiation among adult individuals newly identified as having chronic hepatitis C in metropolitan France, before (i.e., 1 January 2014 to 3 August 2017) and after (4 August 2017 to 31 December 2022) the introduction of universal access to this treatment, univariable and multivariable analyses, Cox proportional hazards models (ANRS FANTASIO 2 study)**

|  | **Univariable analyses** | | | | **Multivariable analyses** | | | |
| --- | --- | --- | --- | --- | --- | --- | --- | --- |
|  | **Before universal access to DAA** | | **After universal access to DAA** | | **Before universal access to DAA** | | **After universal access to DAA** | |
|  | **HR [95% CI]** | ***P-value*** | **HR [95% CI]** | ***P-value*** | **aHR [95% CI]** | ***P-value*** | **aHR [95% CI]** | ***P-value*** |
| **Female sex** | 1.00 [0.98 - 1.03] | 0.714 | 1.04 [1.01 - 1.06] | 0.003 | 0.95 [0.92 - 0.97] | <0.001 | 0.96 [0.93 - 0.98] | <0.001 |
| **Age** (in years) |  |  |  |  |  |  |  |  |
| - Under 30 | 0.27 [0.25 - 0.29] | <0.001 | 0.46 [0.44 - 0.49] | <0.001 | 0.30 [0.28 - 0.33] | <0.001 | 0.48 [0.45 - 0.50] | <0.001 |
| - 30 to 49 | 0.62 [0.60 - 0.63] | <0.001 | 0.85 [0.83 - 0.87] | <0.001 | 0.65 [0.63 - 0.67] | <0.001 | 0.85 [0.83 - 0.87] | <0.001 |
| - 50 to 64 (ref.) | 1 |  | 1 |  | 1 |  | 1 |  |
| - 65 to 74 | 0.99 [0.95 - 1.02] | 0.470 | 0.79 [0.76 - 0.82] | <0.001 | 1.03 [0.99 - 1.07] | 0.100 | 0.93 [0.90 - 0.97] | <0.001 |
| - 75 and over | 0.66 [0.63 - 0.69] | <0.001 | 0.59 [0.56 - 0.62] | <0.001 | 0.70 [0.67 - 0.74] | <0.001 | 0.74 [0.71 - 0.78] | <0.001 |
| **State medical assistance^1^** | 0.81 [0.77 - 0.86] | <0.001 | 1.32 [1.26 - 1.39] | <0.001 | 1.08 [1.02 - 1.14] | 0.011 | 1.57 [1.48 - 1.65] | <0.001 |
| **Complementary state health insurance^2^** | 0.82 [0.79 - 0.84] | <0.001 | 1.10 [1.07 - 1.12] | <0.001 | 0.96 [0.93 - 0.99] | 0.004 | 1.10 [1.07 - 1.13] | <0.001 |
| **Receiving care for alcohol use disorder^3^** | 0.94 [0.91 - 0.98] | 0.005 | 1.06 [1.02 - 1.10] | 0.002 | 0.86 [0.82 - 0.90] | <0.001 | 0.92 [0.88 - 0.96] | <0.001 |
| **Receiving opioid substitution therapy^4^** | 0.85 [0.82 - 0.88] | <0.001 | 1.30 [1.26 - 1.34] | <0.001 | 0.95 [0.92 - 0.99] | 0.012 | 1.21 [1.17 - 1.26] | <0.001 |
| **Prison detention^5^** | 0.54 [0.51 - 0.57] | <0.001 | 0.64 [0.61 - 0.67] | <0.001 | 0.75 [0.70 - 0.80] | <0.001 | 0.55 [0.53 - 0.58] | <0.001 |
| **HBV co-infection^6^** | 0.79 [0.75 - 0.85] | <0.001 | 0.42 [0.39 - 0.45] | <0.001 | 0.67 [0.63 - 0.72] | <0.001 | 0.44 [0.41 - 0.47] | <0.001 |
| **HIV co-infection^6^** | 1.58 [1.53 - 1.63] | <0.001 | 0.77 [0.74 - 0.81] | <0.001 | 1.57 [1.52 - 1.63] | <0.001 | 0.78 [0.75 - 0.82] | <0.001 |
| **Liver disease severity^6^** |  |  |  |  |  |  |  |  |
| - None (ref.) | 1 |  | 1 |  | 1 |  | 1 |  |
| - Fibrosis or compensated cirrhosis | 1.80 [1.73 - 1.86] | <0.001 | 1.20 [1.14 - 1.26] | <0.001 | 1.65 [1.59 - 1.72] | <0.001 | 1.09 [1.03 - 1.15] | <0.001 |
| - Decompensated cirrhosis | 1.74 [1.65 - 1.83] | <0.001 | 1.04 [0.98 - 1.10] | 0.225 | 1.69 [1.61 - 1.78] | <0.001 | 1.02 [0.97 - 1.08] | 0.425 |
| - Hepatocellular carcinoma | 1.30 [1.20 - 1.41] | <0.001 | 0.92 [0.85 - 1.00] | 0.043 | 1.15 [1.06 - 1.26] | 0.001 | 0.83 [0.77 - 0.90] | <0.001 |
| **Psychiatric disorders^6,7^** | 0.94 [0.91 - 0.97] | <0.001 | 1.04 [1.01 - 1.07] | 0.004 | 0.92 [0.89 - 0.94] | <0.001 | 0.97 [0.94 - 1.00] | 0.035 |
| **Chronic renal failure^6^** | 0.50 [0.45 - 0.54] | <0.001 | 0.11 [0.10 - 0.12] | <0.001 | 0.47 [0.42 - 0.51] | <0.001 | 0.10 [0.09 - 0.12] | <0.001 |

(a)HR, (adjusted) hazard ratio; CI, confidence interval; DAA, direct-acting antiviral; HBV, hepatitis B virus; HCV, hepatitis C virus; PAF, population attributable fractions; ref, reference.

^1^ In France, state medical assistance is a measure guaranteeing free access to healthcare for persons with irregular immigration status and limited financial resources.

^2^ State complementary health insurance, free of charge or with a financial contribution, depending on individuals’ financial resources.

^3^ Individuals receiving care for alcohol use disorders during the current or the previous year were identified using a standard algorithm based on alcohol-related hospital claims, treatment dispensation, and long-term disease status during this period (Supplementary Table 1).

^4^ Individuals receiving opioid substitution therapy during the current or the previous year were identified using a standard algorithm based on dispensation of treatment for opioid substitution and/or opioid overdose (buprenorphine, methadone, naltrexone, naloxone) during this period (Supplementary Table 1).

^5^ Individuals who had been detained in prison during the current or previous year were identified through enrollment in the prisoners’ health insurance scheme.

^6^ Individuals with viral co-infections, liver disease severity, psychiatric disorders, or chronic renal failure were identified using standard algorithms based on related hospital claims, laboratory tests, treatment dispensation, and long-term disease status (Supplementary Table 1).

^7^ Psychiatric disorders included mood disorders, psychotic disorders, and other psychiatric disorders.
